# Immune Checkpoint Inhibitor Combination Therapy versus Sunitinib as First-Line Treatment for Favorable-IMDC-Risk Advanced Renal Cell Carcinoma Patients: A Meta-Analysis of Randomized Clinical Trials

**DOI:** 10.3390/biomedicines10030577

**Published:** 2022-03-01

**Authors:** Ray Manneh, Mauricio Lema, Lucía Carril-Ajuria, Linda Ibatá, Susan Martínez, Daniel Castellano, Guillermo de Velasco

**Affiliations:** 1 Medical Oncology Department, Sociedad de Oncología y Hematología del César, Valledupar 200001, Colombia; ray.manneh@sohec.co; 2 Medical Oncology Department, University Hospital 12 de Octubre, 28041 Madrid, Spain; lucia.carril@gustaveroussy.fr (L.C.-A.); daniel.castellano@salud.madrid.org (D.C.); 3 Medical Oncology Department, Clínica de Oncología Astorga, Medellin 050001, Colombia; dirinvestigacion@artoga.com; 4 Medical Oncology Department, Institute Gustave Roussy, 94805 Paris, France; 5 EpiThink Health Consulting—CL 56 A 3 C 42, 10231, Bogota 110111, Colombia; linda.ibata@epithink.com (L.I.); susan.martinez@epithink.com (S.M.)

**Keywords:** renal cell carcinoma, immunotherapy, sunitinib, systematic review, meta-analysis, IMDC favorable-risk group

## Abstract

Background: Novel combination therapies have been shown to improve the outcomes of treatment-naive patients with locally advanced or metastatic renal cell carcinoma (aRCC). However, the optimal systemic therapy for aRCC of favorable risk has yet to be clarified. We aimed to evaluate the efficacy and safety of different immunotherapy (IO) combinations, either with another IO (IO–IO) or with an antiangiogenic (IO–TKI), versus sunitinib in the first-line setting in aRCC patients with favorable IMDC risk. Methods: We conducted a systematic search for evidence in PubMed, Ovid MEDLINE, Embase, and the Cochrane Central Register of Controlled Trials published up to February 2021. The GRADE approach was used to assess the quality of evidence. Survival hazard ratios were extracted for analysis in the favorable-risk aRCC subgroup (IMDC). A sensitivity analysis was performed excluding trials of combination therapy without TKI. Results: Five randomized controlled phase III trials with a total of 1088 patients were included in the analysis. The studies compared different combinations versus sunitinib monotherapy. All clinical trials reported overall survival (OS), progression-free survival (PFS), and objective response rate (ORR) data. Four out of five trials reported complete response (CR). There was no difference in OS nor PFS between treatment arms in the IMDC favorable-risk subgroup analysis (OS: HR = 1.07, 95% CI = 0.81–1.41; PFS: HR = 0.74, 95% CI = 0.46–1.19). A benefit in ORR and CR was found for combination therapy vs. sunitinib (ORR: HR = 1.89, 95% CI = 1.29–2.76; CR: HR = 3.58, 95% CI = 2.04–6.28). In the sensitivity analysis, including only IO–TKI vs. sunitinib, no difference in OS was found; however, an advantage in PFS was observed (OS: HR = 0.99, 95% CI 0.69–1.43; PFS: HR = 0.60 (0.45–0.81). The safety profile reported is consistent with previous reports. We did not find differences in the incidence of any adverse event (AE) or of grade ≥3 AEs. Conclusion: This meta-analysis shows that combinations of IO–KI as first-line treatment in favorable-IMDC-risk aRCC improve PFS, ORR, and CR, but not OS, versus sunitinib.

## 1. Introduction

Kidney cancer is the cause of 2.2% of all cancers globally, with ~431,288 cases reported in 2020 [1]. The age-standardized kidney cancer rate for both sexes is 4.4 per 100,000, with a cumulative risk (0 to 74 years) of 0.51%. Incidence, prevalence, and mortality vary significantly by geographic region, with the highest numbers in North America, where it is among the top 10 causes of cancer [2]. Renal cell carcinoma accounts for 90% of all kidney cancers. Risk assessment is essential for stratifying patients for therapeutic orientation and determining prognosis. Five-year survival rates are 80–90% among stage I or II patients at diagnosis, and around 16% in metastatic disease [3].

In recent years, tyrosine kinase inhibitors targeting vascular endothelial growth factor (VEGF) have been the standard of care for patients with locally advanced or metastatic renal cell cancer (aRCC) [4]. However, according to the subtypes, histological characteristics, cytogenetics, and molecular markers, the treatment response can be variable [5], and long-term remission is scarce. Recent studies have shown the potential of new therapies to improve aRCC patients’ prognosis, including the dual immune checkpoint inhibitor combination (IO–IO) nivolumab plus ipilimumab (NIVO + IPI), as well as immunotherapy–tyrosine kinase inhibitor combinations (IO–TKI), with numerous novel regimens under investigation [6]. To select treatment strategies, current evidence has shown that it is important to stratify patients according to their risk situation using the International mRCC Database Consortium Prognostic Model (IMDC score).

Patients with favorable risk are defined by the absence of any of the following risk factors: Karnofsky performance status less than 80%, time from diagnosis to treatment less than 1 year, hemoglobin concentration less than the lower limit of normal (LLN), serum calcium more than the upper limit of normal (ULN), neutrophil count more than the ULN, and platelet count more than the ULN [7]. 

The options available for the management of aRCC with favorable IMDC risk are extremely variable, from active surveillance to combinations of systemic therapy. Active surveillance may be recommended for selected, asymptomatic patients with a low volume disease burden [8]. TKI monotherapy provides great control of disease, reaching a median OS of ~29 months, and is recognized by the international guidelines (sunitinib, pazopanib, or tivozanib) as an adequate treatment option that physicians could consider in selected aRCC patients [9,10].

However, combinations of IO–TKI are the preferred regimens in frontline settings, according to the NCCN and ESMO guidelines. Moreover, in addition to the NIVO–IPI and pembrolizumab–axitinib (PEMBRO–AXI) combinations, another two immunotherapy combinations have become standards of care in first-line settings: pembrolizumab–lenvatinib (PEMBRO–LENVA), and nivolumab–cabozantinib (NIVO–CABO). These two combinations have been added to the preferred regimens in frontline settings by the NCCN guidelines [9,10].

Despite the high amount of different treatment alternatives in first-line settings, their benefit in favorable-risk aRCC is still not well established. Therefore, we conducted a meta-analysis of available randomized clinical trials comparing IO combinations versus sunitinib in frontline settings, in order to study the efficacy and safety of IO combinations compared to sunitinib alone in favorable-risk aRCC patients. 

## 2. Materials and Methods

This systematic review was conducted according to the Cochrane Handbook for Systematic Reviews of Interventions to pool the evidence [11]. The results of this study are reported following the Preferred Reporting Items for Systematic Reviews and Meta-Analyses report (PRISMA Statement) guidelines [12]. The protocol was registered on PROSPERO (CRD42022300758).

### 2.1. Eligibility Criteria

Type of Study Design Included: Phase III randomized clinical trials (RCTs) were eligible for inclusion. No language, publication date, or publication status restrictions were imposed. 

Types of Participants: The study population consisted of favorable-risk aRCC patients treated with frontline therapy within clinical trials comparing IO combinations versus sunitinib. 

Types of Interventions Included: The agents consisted of the following immunotherapy combinations (experimental arms): NIVO–IPI, PEMBRO–AXI, avelumab–axitinib (AVELU–AXI), PEMBRO–LENVA, and NIVO–CABO, along with sunitinib (control arm), given in a frontline setting. 

Types of Outcome Measures Included: The primary outcomes were (1) overall survival (OS), (2) progression-free survival (PFS), and (3) incidence of grade ≥3 AEs according to the Common Terminology Criteria for Adverse Events (CTCAE) version 4. 

### 2.2. Search Strategy

A comprehensive search of PubMed, EMBASE, and the Cochrane Library for related studies published before February 2021 was performed. Additionally, http://clinicaltrials.gov, (accessed on 10 March 2021) abstracts, and virtual meeting presentations containing the same terms, from the American Society of Clinical Oncology (ASCO) and the European Society of Medical Oncology (ESMO) conferences held between January 2015 and February 2021, were also used to identify relevant and ongoing clinical trials. We used (‘carcinoma’, ‘renal cell’ OR ‘RCC-derived cell line’ AND ‘metastatic cancer’) AND (‘Immunotherapy’) AND (‘sunitinib’) as a search algorithm (see Supplementary Materials). 

### 2.3. Data Collection

Two independent investigators reviewed the publications and extracted the data (R.M. and M.L.); disagreements were resolved by consensus. All citations found during the searches were stored in a reference database. The following data were extracted: author, demographic data, treatment regimens, sample size, and summary estimates of interest outcomes. The outcomes of interest were overall survival (OS), progression-free survival (PFS), objective response rate (ORR), complete response (CR), and incidence of grade ≥3 adverse events (AEs). 

### 2.4. Data Analysis

We performed a direct frequentist meta-analysis using a random-effects model [13]. Authors decided whether patient and treatment characteristics, time of follow-up, and outcome definitions were sufficiently similar for meta-analysis. We used HR and 95% CI, presented as forest plots. We then synthesized the HR data across studies using the random-effects (DerSimonian and Laird) model to obtain pooled effect sizes [14]. For incidence of any grade AE and grade ≥3 AEs, a pooled relative risk was calculated.

The presence of statistical heterogeneity was first assessed using Cochran’s Q test (considered significant for *p* < 0.05) and quantified using I2 statistics [15]. A sensitivity analysis was performed by recalculating the pooled HR estimate for trials where the intervention included a TKI (nivolumab plus ipilimumab was excluded). This analysis intends to determine whether the pooled estimates vary when checkpoint inhibitors are not included.

Finally, potential publication bias was evaluated using Egger’s test [16] to examine individual study estimates’ relative symmetry around the overall estimate. A two-tailed *p*-value of less than 0.05 was considered statistically significant. All statistical analyses were performed using Stata version 12.0 software (StataCorp., College Station, TX, USA).

We assessed the methodological quality of the eligible trials using Cochrane’s risk of bias (RoB) tool on a three-point scale: high bias, low bias, and unclear [17]. The quality of evidence was rated according to GRADE methods as high, moderate, low, or very low, based on the risk of bias, directness, precision, and consistency in treatment effects. A high-quality evidence level was assigned to well-designed RCTs with consistent findings (I2 < 50%). The quality of evidence was downgraded to moderate if at least one of the four criteria was not met, and it was downgraded to low if two or more criteria were not met. We concluded a high risk of bias in the body of evidence if at least one RCT had a high risk of bias. The body of evidence was downgraded when we suspected a high risk of publication bias due to the unavailability of the results on ClinicalTrials.gov or in journal articles (see Supplementary Materials) [18].

## 3. Results

### 3.1. Characteristics of Trials, Patients, and Interventions

**Figure 1:**
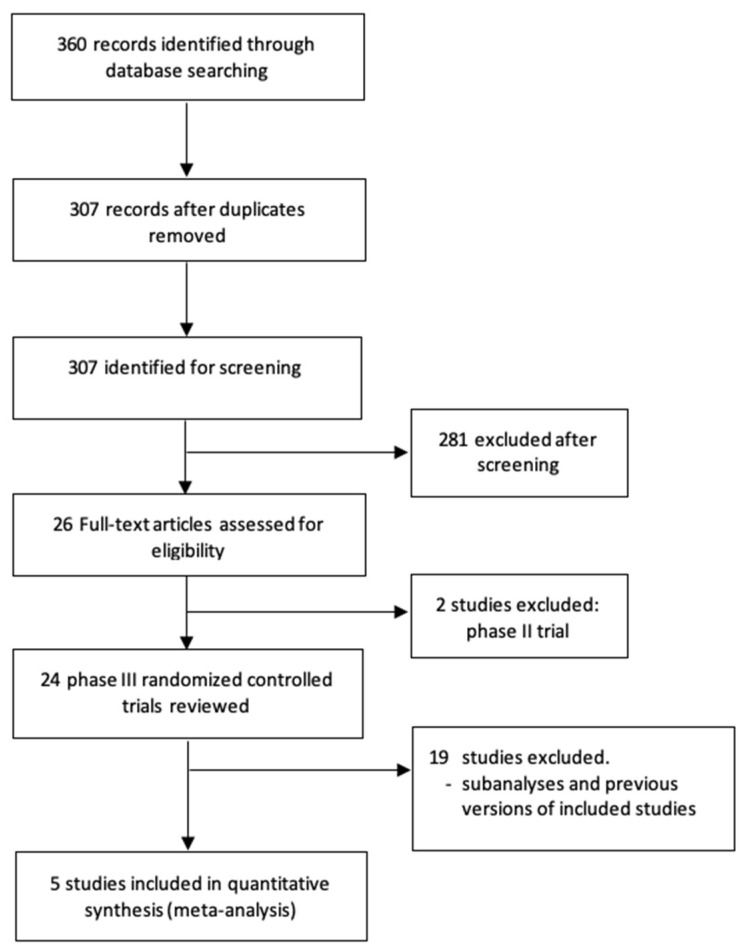
Flow diagram of the systematic review.

A total of 360 potentially relevant records were identified from electronic databases. Based on the inclusion and exclusion criteria described, five randomized trials [19,20,21,22,23] were included in this review (Preferred Reporting Items for Systematic Reviews and Meta-Analyses diagram in Figure 1). The selected studies included 1088 patients with favorable-risk aRCC—541 patients randomized to combination therapy and 547 patients to sunitinib. Table 1 shows the main characteristics of the included trials. 

Included studies were open-label RCTs in adults with previously untreated advanced or metastatic RCC with a clear-cell component or sarcomatoid features, and with measurable disease according to the Response Evaluation Criteria in Solid Tumors (RECIST), version 1.1, and in the IMDC favorable-risk group. The combination therapy regimens differed between the trials. The comparison arm in all studies was orally administered sunitinib. Treatments continued until documented disease progression or unacceptable toxicity, withdrawal of consent, or the study’s end. Patient characteristics were well balanced between the treatment arms in all included studies. 

### 3.2. Quality Assessment

The selected evidence was evaluated using the Cochrane Collaboration tool [17]. The risk of bias due to the lack of blinding allocation was not considered because the intravenous placebo was impractical, and it did not impact the outcomes evaluated. The quality assessment resulted in a low risk of bias for the included studies, but the body of evidence for some outcomes was downgraded because of inconsistency and imprecision (see Supplementary Materials).

### 3.3. Efficacy

**Figure 2:**
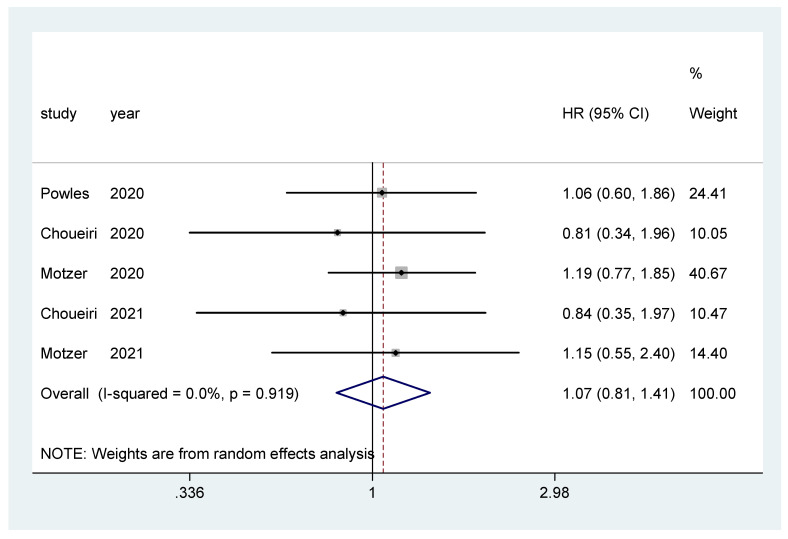
Forest plot estimating OS in comparison of combined treatment versus sunitinib in the favorable-risk group. HR: hazard ratio; CI: confidence interval.

Overall survival was the primary endpoint in two studies [20,22], and secondary outcome in the other three [19,21,23]. The pooled hazard ratio (HR) for OS in the favorable-risk subgroup did not show statistically significant differences between the evaluated treatments (HR = 1.07, 95% CI = 0.81–1.41; I2 = 0.0%) (Figure 2). There was no evidence of publication bias (Egger’s test, *p* = 0.068).

**Figure 3:**
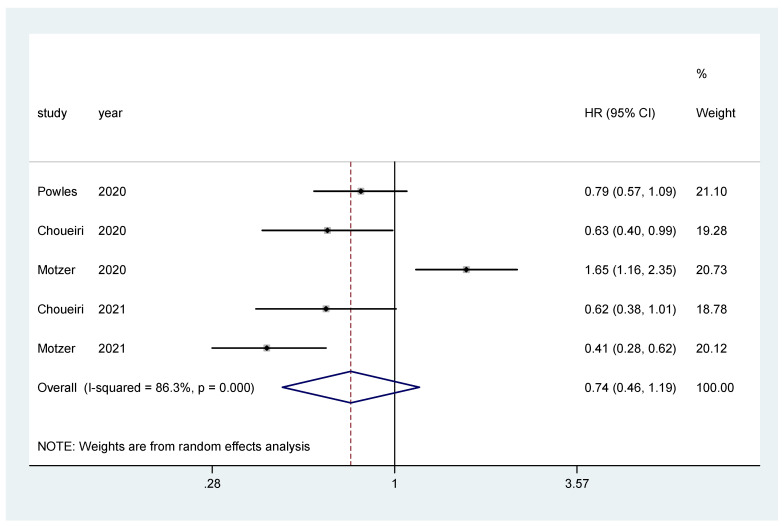
Forest plot estimating PFS in comparison of combined treatment versus sunitinib in the favorable-risk group. HR: hazard ratio; CI: confidence interval.

The pooled hazard ratio (HR) for PFS in the favorable-risk subgroup showed no statistically significant differences between the evaluated treatments (HR = 0.74, 95% CI = 0.46–1.19), with high heterogeneity (I2 = 86.3%) (Figure 3); for PFS there was no evidence of publication bias (Egger’s test, *p* = 0.437).

**Figure 4:**
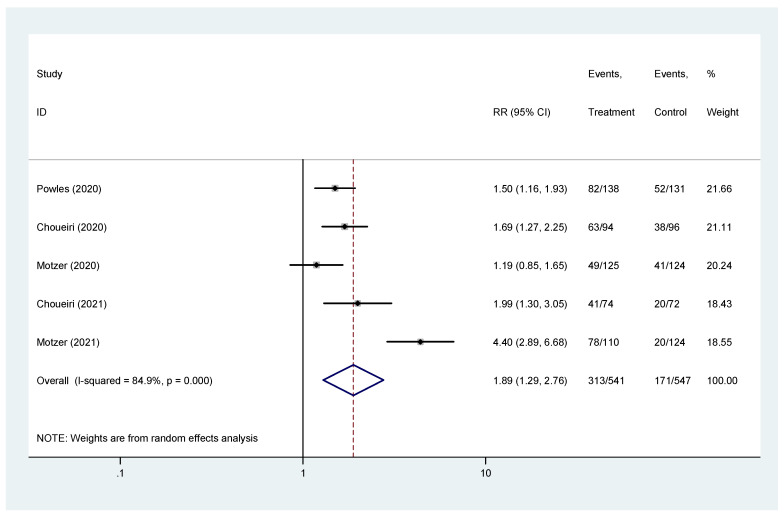
Forest plot estimating ORR in comparison of combined treatment versus sunitinib in the favorable-risk group. HR: hazard ratio; CI: confidence interval.

The pooled hazard ratio (HR) for ORR in the favorable-risk subgroup showed statistically significant differences between the evaluated treatments (HR = 1.89, 95% CI = 1.29–2.76), with high heterogeneity (I2 = 84.9%) (Figure 4); for ORR there was no evidence of publication bias (Egger’s test, *p* = 0.845).

**Figure 5:**
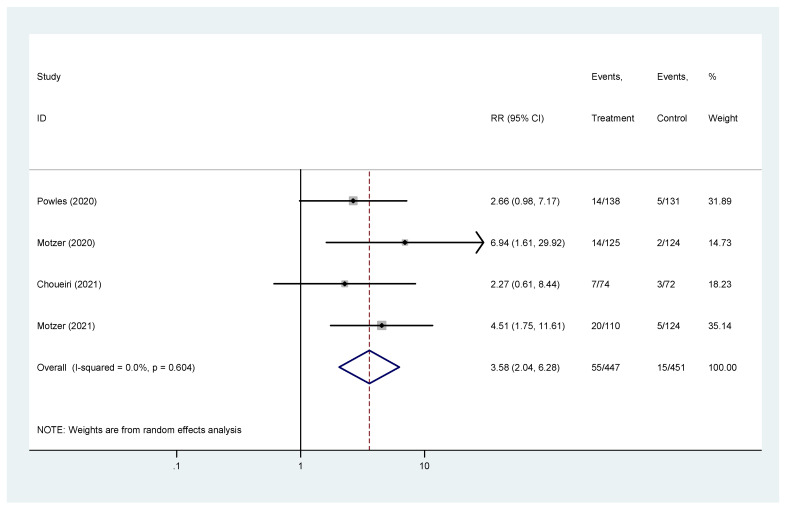
Forest plot estimating CR in comparison of combined treatment versus sunitinib in the favorable-risk group. HR: hazard ratio; CI: confidence interval.

Complete response was reported in four studies [19,20,22,23]. The pooled hazard ratio (HR) for CR in the favorable-risk subgroup showed statistically significant differences between the evaluated treatments (HR = 3.58, 95% CI = 2.04–6.28), without heterogeneity (I2 = 0%) (Figure 5); for CR there was no evidence of publication bias (Egger’s test, *p* = 0.469).

### 3.4. Sensitivity Analysis

**Figure 6:**
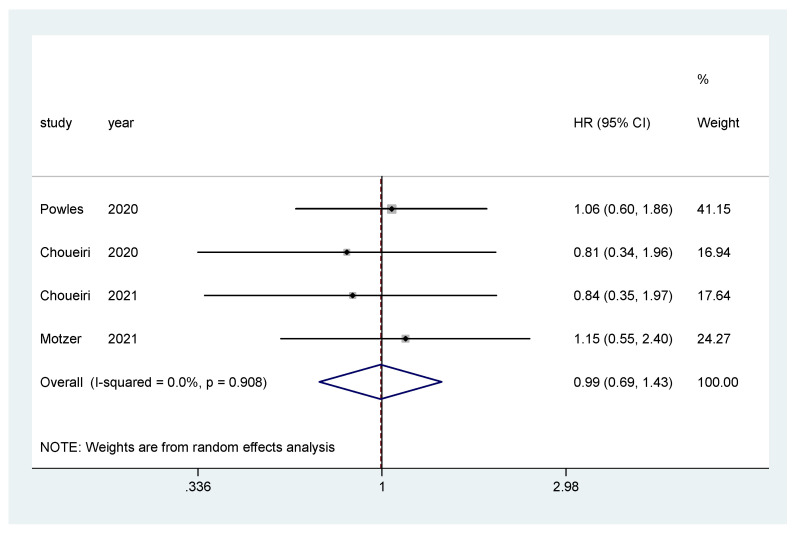
Forest plot estimating OS in comparison of TKI combined treatment versus sunitinib in the favorable-risk group. HR: hazard ratio; CI: confidence interval.

Additionally, subgroup analysis was performed only for IO–TKI combinations (nivolumab plus ipilimumab was excluded) for OS and PFS. The pooled hazard ratio (HR) for OS in the favorable-risk subgroup did not show statistically significant differences between the evaluated treatments (HR = 0.99, 95% CI = 0.69–1.43; I2 = 0.0%) (Figure 6). There was no evidence of publication bias (Egger’s test, *p* = 0.274).

**Figure 7:**
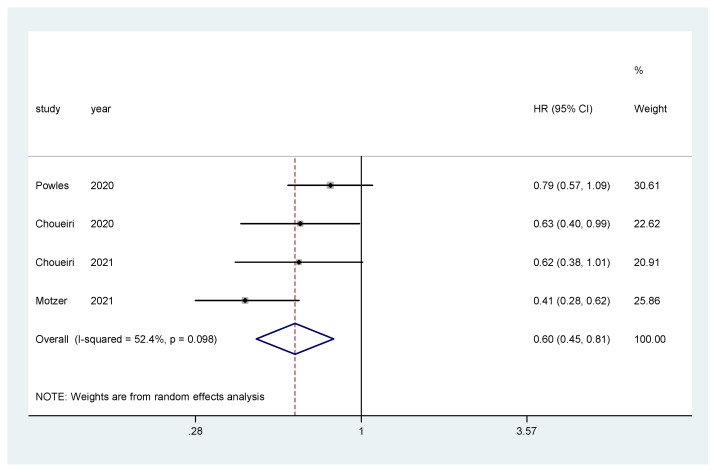
Forest plot estimating PFS in comparison of TKI combined treatment versus sunitinib in the favorable-risk group. HR: hazard ratio; CI: confidence interval.

The pooled hazard ratio (HR) for PFS in the favorable-risk subgroup showed statistically significant differences between the evaluated treatments (HR = 0.60, 95% CI = 0.45–0.81), with moderate heterogeneity (I2 = 52.4%) (Figure 7) and no publication bias (Egger’s test, *p* = 0.592).

### 3.5. Safety

In this section we report AEs for all treated patients in the included clinical trials. There is no available data on AEs specifically for patients in the favorable-risk group. Treatment-related AEs occurred in similar proportions in the combination therapy and sunitinib arms in all included studies (91–99.5% and 93–99.3%, respectively). Table 2 presents grade ≥3 AEs reported in patients in either arm; it includes events reported between the first dose and 30 days after the last dose of the studied therapy. 

**Figure 8:**
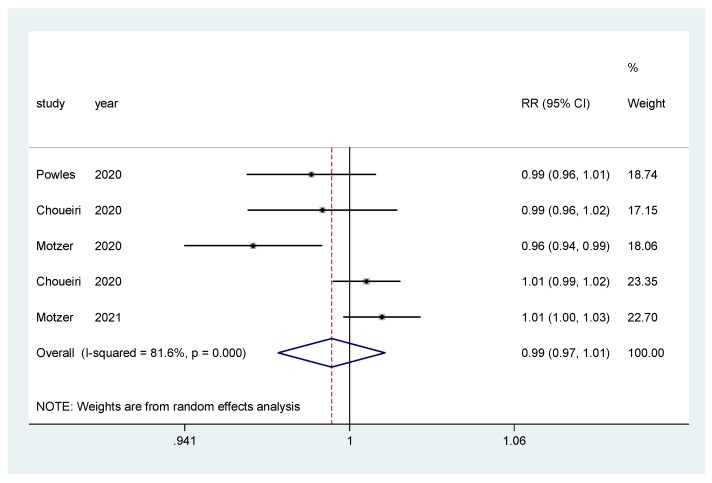
Forest plot estimating the pooled RR of any AEs for IO combination treatment versus sunitinib.

**Figure 9:**
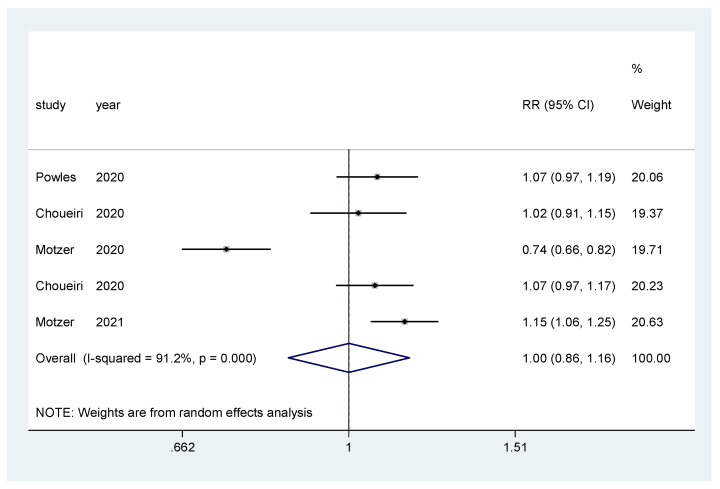
Forest plot estimating the pooled RR of grade ≥3 AEs for IO combination treatment versus sunitinib.

For incidence of any grade AE and of grade ≥3 AEs, a pooled relative risk was calculated. There was no difference in the incidence of any grade AEs (RR = 0.99, CI 95% = 0.97–1.01) (Figure 8), nor in the incidence of grade ≥3 AEs (RR = 1.00, CI 95% = 0.86–1.16) (Figure 9).

## 4. Discussion

In the era of new therapies for advanced or metastatic RCC, risk assessment is established using the International Metastatic Renal Cell Carcinoma Database (IMDC) criteria. Although the MSKCC classification can also be used, the IMDC risk score was developed using patients treated with targeted therapy—in contrast to the MSKCC classification, which used data from patients receiving cytokine therapy—and it is currently used in most pivotal immuno-oncology trials [24,25]. Stratification has become a crucial part of clinical and therapeutic decision making for these patients, and the efficacy of new agents in each subgroup of patients is an aspect of particular interest. Massari et al. confirmed the survival benefit of IO–TKI combination therapies compared to sunitinib in a recent meta-analysis [26]. Our aim was to conduct a systematic review of studies of advanced mRCC to determine the effect of combination therapy in the IMDC favorable-risk group. Five phase III RCTs with different combination therapy regimens (NIVO–IPI, PEMBRO–AXI, AVELU–AXI, PEMBRO–LENVA, and NIVO–CABO) were included, and all were compared with sunitinib at a standard dose.

CheckMate 214 showed a survival benefit for patients with intermediate- and low-risk mRCC treated with NIVO–IPI versus sunitinib, with a follow-up of four years [27,28]. However, the survival results were not conclusive in the favorable-risk-group patients [29]. JAVELIN Renal 101 and KEYNOTE-426 demonstrated the survival superiority of AVELU–AXI and PEMBRO–AXI compared to oral sunitinib in patients with aRCC; however, for the favorable-risk subgroup, JAVELIN Renal 101 reported a statistically significant improvement in PFS versus sunitinib, but the OS data were still immature [21]. KEYNOTE-426 did not show survival advantages of PEMBRO–AXI versus sunitinib in this group [22]. CheckMate 9ER [30] results showed the superiority of NIVO–CABO versus sunitinib in terms of OS, PFS, and objective response rate in the first-line treatment of patients with aRCC. Although the favorable-risk group sub-analysis showed a promising trend for the combination therapy, the results were not statistically significant. The CLEAR trial evaluated lenvatinib in combination with pembrolizumab or everolimus in the treatment of advanced renal cell carcinoma. This study showed that PEMBRO–LENVA was associated with significantly longer PFS and OS than sunitinib; however, it did not show an OS benefit in the favorable-risk population [23].

All of the treatments’ safety reports were consistent with previous trials, including the proportion of treatment-related events, adverse events leading to discontinuation, and selected adverse events (potentially immune-mediated).

The results of this meta-analysis focusing on favorable-risk mRCC show that IO–TKI combination improves PFS, ORR, and CR, but not OS. Current guidelines recommend IO–TKI combinations as preferred options for IMDC favorable-risk patients [9,10]; however, careful selection of treatment should be assessed based on these data. In fact, combination therapies such as bevacizumab–atezolizumab did not continue being developed due to their lack of impact in OS; thus, it should be clear that in this specific subgroup of favorable-risk patients, IO–TKI combinations have not proven to increase OS [31]. Financial burden and toxicity issues are factors that should be taken into account when deciding the best treatment for our patients, especially in this subgroup of favorable-risk patients. So far, the IMDC risk score model remains the only validated prognostic score for aRCC patients treated with systemic therapy, and it is the only tool used to guide frontline treatment selection [32]. Unfortunately, other promising potential biomarkers of response—such as PD-L1 expression or tumor mutational burden (TMB)—have failed to demonstrate a predictive role in aRCC [33]. Hopefully, molecular classification may help in the future to identify which patients may benefit from combination therapy or TKI alone and, thus, to better select the best treatment strategy for our patients. 

The advantage of combination therapy versus sunitinib in the first-line management of advanced or metastatic RCC patients is well known [34]; however, it should be highlighted that these benefits were not observed in the sub-analysis of patients with favorable IMDC risk. Our meta-analysis also failed to demonstrate any advantage in terms of OS. Nevertheless, after performing a sensitivity analysis including only IO–TKI trials, a benefit in PFS was observed for the combination arms compared to sunitinib alone (HR = 0.60, 95% CI = 0.45–0.81); there was no difference between treatments in terms of OS. These findings suggest that treatment selection in favorable-risk patients should be made carefully. As no advantage in OS has been demonstrated for the IO combinations compared to sunitinib in favorable-risk patients, treatment selection in these patients should be done carefully, as other factors—such as the toxicity profile, drug availability, financial issues, and the patients’ preferences—will be especially relevant in the treatment choice for this subgroup. 

Although the risk of bias of these studies individually was relatively low, the fact that our analysis focused on summary estimators, and not individual data, is a limitation that should be taken into account when interpreting our results. It is essential to highlight the high heterogeneity of PFS across studies regarding the favorable-risk population—probably due to the different definitions of this endpoint between the trials. The small size of the favorable-risk subgroups, the short follow-up time, and the reporting of this sub-analysis in only five RCTs probably constitute significant limitations in demonstrating differences in efficacy between the evaluated treatments.

In conclusion, combination therapy—either IO–IO or IO–TKI—has become the new standard of care in frontline settings in aRCC after demonstrating its superiority compared to sunitinib alone. However, this benefit is still not clear in patients with a favorable risk. Our results suggest a benefit in PFS from IO–TKI compared to sunitinib in this population, but not in OS. Treatment selection should be made carefully in favorable-risk patients, taking into account other factors that may influence treatment decisions. More prospective trials with a larger sample size and longer-term follow-up are needed in order to better establish the impact on OS of combination therapies compared to sunitinib alone in favorable-risk aRCC patients. 

## Figures and Tables

**Table 1:** Characteristics of trials comparing combination therapy vs. sunitinib in first-line treatment for advanced renal cell carcinoma.

Author (Year)/Study	Population	Intervention Regimen, n	Comparator Regimen, n	Median Follow-Up	Median OS and PFS
Choueiri et al., 2021 [19] CheckMate 9ER	Previously untreated advanced or metastatic RCC, clear-cell component, or sarcomatoid features.	**Nivolumab** 240 mg flat dose IV every two weeks (not exceeding a total of two years from cycle 1) + **cabozantinib** 40 mg orally once daily (may continue beyond two years) n = 74	Sunitinib 50 mg orally for 4 weeks (6-week cycles) beyond two years in the absence of progression or unacceptable toxicity, n = 72	18.1 months	PFS NIVO + CABO: 24.7 months SUN: 12.8 months OS NIVO + CABO: not reached SUN: not reached
Motzer et al., 2020 [20] CheckMate214	Advanced or metastatic RCC patients with a clear-cell component.	**Nivolumab** 3 mg/kg plus **ipilimumab** 1 mg/kg IV every three weeks for four doses (induction) followed by Nivolumab 3 mg/kg IV every two weeks (maintenance) n = 125	Sunitinib 50 mg orally, once per day, for 4 weeks on and 2 weeks off each 6-week cycle. A maximum of two dose reductions was permitted in 12.5 mg increments per day (the daily dose must have been ≥25 mg)/n = 124	43.6 months in the NIVO + IPI arm and 32.3 months in the SUN arm	PFS NIVO + IPI: 17.0 months SUN: 28.8 months OS NIVO + IPI: not reached SUN: not reached
Choueiri et al., 2020 [21] JAVELIN Renal 101	Untreated advanced renal cell carcinoma patients with a clear-cell component.	**Avelumab** 10 mg/kg as 1 h IV infusion every two weeks, plus **axitinib** orally at a starting dose of 5 mg twice daily on a continuous dosing schedule, n = 94	Sunitinib was administered at a dose of 50 mg orally once daily for 4 weeks of a 6-week cycle, n = 96	19.3 months in the combination arm and 19.2 months in the sunitinib arm.	PFS AVELU + AXI: 24.0 months SUN:16.7 months OS AVELU + AXI: not reached SUN: not reached
Powles et al., 2020 [22] Keynote 426	Newly diagnosed or recurrent stage IV clear-cell renal cell carcinoma; had received no previous systemic therapy for advanced disease.	**Pembrolizumab** IV 200 mg once every three weeks (maximum of 35 cycles), plus **axitinib** orally at a dose of 5 mg twice daily, with dose adjustments according to safety criteria, n = 138	Sunitinib orally 50 mg daily for the first 4 weeks of each 6-week cycle; the dose could be reduced to 37.5 mg, then 25 mg, for the first 4 weeks of each 6-week cycle to manage toxic effects, n = 131	30.6 months	PFS PEMBRO + AXI: 20.7 months SUN: 17.8 months OS PEMBRO + AXI: 72.3 months SUN: 73 months
Motzer et al., 2021 [23] CLEAR	Patients with advanced renal cell carcinoma and no previous systemic therapy	**Lenvatinib** (20 mg orally once daily) **plus pembrolizumab** (200 mg intravenously once every 3 weeks); **lenvatinib** (18 mg orally once daily) **plus everolimus** (5 mg orally once daily), n = 110	Sunitinib (50 mg orally once daily, alternating 4 weeks receiving treatment and 2 weeks without treatment), n = 124	26.6 months	PFS LENVA + PEMBRO: 28.1 months SUN: 12.9 months OS LENVA + PEMBRO: not reached SUN: not reached

RCC: renal cell carcinoma; IMDC: International Metastatic RCC Database Consortium; IV: intravenous; RECIST: Response Evaluation Criteria in Solid Tumors; IQR: interquartile range; CI: confidence interval; NIVO: nivolumab; CABO: cabozantinib; SUN: sunitinib; IPI: ipilimumab; AVELU: avelumab; AXI: axitinib; PEMBRO: pembrolizumab.

**Table 2:** Grade ≥3 AEs that were reported in patients included in trials comparing combination therapy vs. sunitinib in first-line treatment for advanced renal cell carcinoma.

	CheckMate 9ER		CheckMate 214		Javelin101		Keynote426		CLEAR	
Events	NIVO + CABO	SUN	NIVO+	SUN	AVELU + AXI	SUN	PEMBRO + AXI	SUN	LEN + PEMBRO	SUN
(%)			IPI							
	N = 320	N = 320	N = 547	N = 535	N = 434	N = 439	N = 429	N = 425	N = 352	N = 340
Treatment-related AEs	61%	51%	47.3%	64.1%	71.2%	71.5%	67%	62%	71.6%	58.8%
Diarrhea	6.9%	4.4%	3.8%	5.8%	6.7%	2.7%	10.7%	5%	9.7%	5.3%
Nausea	0.6%	0.3%	1.5%	1.3%	1.4%	1.6%	0.5%	0.9%	2.6%	0.6%
Stomatitis	2.5%	2.2%	0%	2.6%	1.8%	0.9%	1%	2.1%	1.7%	2.1%
Mucosal inflammation	0.9%	2.5%	0.2%	2.8%	1.2%	1.1%	0.9%	1.6%	NR	NR
Increased lipase	6.2%	4.7%	10.6%	6.7%	NR	NR	NR	NR	12.8%	8.8%
Decreased appetite	1.9%	1.2%	1.3%	1.1%	2.1%	0.9%	2.1%	0.5%	4.0%	1.5%
Decreased weight	0.6%	0%	NR	NR	2.8%	0.9%	3.0%	0.2%	8.0%	0.3%
Back pain	1.6%	1.9%	NR	NR	0.5%	1.8%	0.9%	1.6%	1.1%	2.1%
Asthenia	4.4%	3.1%	1.8%	2.4%	2.5%	3.0%	1.4%	3%	4.5%	3.2%
Vomiting	1.9%	0.3%	0.7%	1.9%	0.9%	1.6%	0.2%	1%	3.4%	1.5%
Dyspnea	NR	NR	NR	NR	3.0%	1.6%	1.6%	1.2%	2.6%	2.4%
Fatigue	3.4%	4.7%	4.4%	9.7%	3.5%	3.6%	3%	5%	4.3%	4.4%
Arthralgia	0.3%	0.3%	NR	NR	0.9%	0.5%	0.7%	0.5%	1.4%	0.3%
Rash	1.9%	0%	1.6%	0%	0.5%	0.5%	0.2%	0.2%	3.7%	0.6%
Hypertension	12.5%	13.1%	0.7%	17.0%	25.6%	17.1%	22%	20%	27.6%	18.8%
Palmoplantar erythrodysesthesia	7.5%	7.5%	0.2%	9.3%	5.8%	4.3%	0.5%	5%	4.0%	3.8%
Anemia	1.9%	3.8%	0.5%	4.3%	1.6%	8.2%	0.2%	4.0%	2.0%	5.3%
Thrombocytopenia	0.6%	4.7%	0%	4.3%	0.2%	6.2%	0%	5.2%	0.6%	5.6%
AST increased	3.4%	1.2%	NR	NR	3.9%	2.1%	6.8%	1.6%	3.1%	0.9%
ALT increased	5.3%	2.2%	NR	NR	6.0%	2.5%	13%	2.6%	4.3%	2.4%
Proteinuria	2.8%	2.2%	NR	NR	NR	NR	3%	3%	7.7%	2.9%
Treatment-related AE leading to discontinuation	NR	NR	15.4%	7.3%	7.6%	13.4%	18.2%	16.2%	NR	NR
Treatment-related deaths	1% ^a^	2% ^b^	NR	NR	0.3% ^c^	0.2% ^d^	0.9% ^e^	1.6% ^f^	NR	NR

NIVO: nivolumab; CABO: cabozantinib; SUN: sunitinib; IPI: ipilimumab; AVELU: avelumab; AXI: axitinib; PEMBRO: pembrolizumab; AE: adverse event. N: total number of patients with available safety data information. Includes events reported between the first dose and 30 days after the last dose of the studied therapy. Listed are grade ≥3 AEs that were reported in >1% of the patients in either arm. ^a^ Small intestine perforation; ^b^ pneumonia, respiratory distress; ^c^ intervention group was attributed to sudden death, myocarditis, and necrotizing pancreatitis; ^d^ intestinal perforation; ^e^ one patient each with myasthenia gravis, myocarditis, necrotizing fasciitis, and pneumonitis; ^f^ one patient each with acute myocardial infarction, cardiac arrest, gastrointestinal hemorrhage, hemorrhage intracranial, hepatitis fulminant, malignant neoplasm progression, and pneumonia.

## Supplementary Materials

The following supporting information can be downloaded at: https://www.mdpi.com/article/10.3390/biomedicines10030577/s1, Figure S1: Risk of bias summary of RCTs with Cochrane Collaboration tool, Figure S2: Risk of bias summary of RCTs with Cochrane Collaboration tool, Table S1: GRADE table for survival from clinical trial data.

## Abbreviations

**Abbreviations/Acronyms**	**Definition**
aRCC	Advanced renal cell carcinoma
IO	Immunotherapy
IO–IO	Immunotherapy combination
IO–TKI	Immunotherapy–VEGFR tyrosine kinase inhibitor combination
IMDC	International Metastatic RCC Database Consortium
OS	Overall survival
ORR	Objective response rate
PFS	Progression-free survival
AEs	Adverse events
CR	Complete response
HR	Hazard ratio
LLN	Lower limit of normal
ULN	Upper limit of normal
NCCN	National Comprehensive Cancer Network
ESMO	European Society of Medical Oncology
PEMBRO–AXI	Pembrolizumab–axitinib
PEMBRO–LENVA	Pembrolizumab–lenvatinib
NIVO–CABO	Nivolumab–cabozantinib
PRISMA Statement	Preferred Reporting Items for Systematic Reviews and Meta-Analyses report
RCT	Randomized clinical trial
CTCAE	Common Terminology Criteria for Adverse Events version 4
ASCO	American Society of Clinical Oncology
ROB	Risk of bias
RECIST	Response Evaluation Criteria in Solid Tumors
IV	Intravenous
IQR	Interquartile range
N	Number of patients
TMB	Tumor mutational burden
