# Supplementary material for: Immune Checkpoint Inhibitor Combination Therapy versus Sunitinib as First-Line Treatment for Favorable-IMDC-Risk Advanced Renal Cell Carcinoma Patients: A Meta-Analysis of Randomized Clinical Trials
Source: Biomedicines. 2022 Mar 1;10(3):577. doi: 10.3390/biomedicines10030577 (PMC8945232; doi:10.3390/biomedicines10030577)
Supplement: Supplementary file 1 [file biomedicines-10-00577-s001.zip › biomedicines-1534158-supplementary.pdf]

Supplementary Materials

Combination of TKI and immune checkpoint inhibitors versus standard of care in first-line treatment for advanced renal cell carcinoma of favorable risk patients A meta-analysis of randomized clinical trials

Contents

1. Search strategies
2. Risk of bias assessments
3. GRADE evidence profiles

Section S1. Search strategies.

The search strategy was developed with the support of two expert epidemiologists with experience in systematic reviews (LI, SM). Electronic searches of registered databases were conducted in December 2020 and updated in February 2021. Searches were conducted to identify randomised controlled trials examining Immunotherapy and sunitinib in first-line treatment for advanced renal cell carcinoma in favourable risk patients. Medline (PubMed), Embase, CENTRAL (OVID) and ClinicalTrials were searched for clinical trials published up till February 2021, which met the predetermined inclusion criteria. Thematic experts provided feedback to the search strategy. The strategies combined medical subject headings (MeSH) and keywords for the terms: "Carcinoma, Renal Cell" OR "RCC-derived cell line" AND "metastatic cancer" AND "Immunotherapy" AND "sunitinib".
Hand searches were performed to identify any studies not previously included.

Medline (PubMed)
#1 ((((((((Carcinoma, Renal Cell[MeSH Terms]) OR (kidney carcinoma)) OR (adenocarcinoma, kidney)) OR (carcinoma, kidney)) OR (kidney hypernephroma)) OR (rcc-derived cell line)) OR (hypernephroid carcinoma cell line)) OR (kidney carcinoma cell line)) OR (renal carcinoma cell line)) OR (Renal cancer) 146,761
#2 (((((cancer metastasis) OR (metastasic type)) OR (metastatic cancer)) OR (metastatic carcinoma)) OR (metastatic disease)) OR (metastatic tumor[MeSH Terms]) 532,172
#3 Search #1 AND #234,691
#4 (((((((((Immunotherapy[MeSH Terms]) OR (pembrolizumab)) OR (nivolumab)) OR (avelumab)) OR (ipilimumab)) OR (Atezolizumab)) OR (bevacizumab)) OR (protein tyrosine kinase inhibitor)) OR (protein tyrosine kinase inhibitor)) OR (tyrosine kinase inhibitor)) OR (tyrosine protein kinase inhibitor) 405,413
#5 ((sunitinib) OR (sunitinib)) OR (sunitinib malate) 6,349
#6 Search #4 AND #5 3,387
#7 Search #3 AND #6 1,196
Search #7 Filters: Clinical Trial, 100

Embase
#1 ('renal cell carcinoma'/mj OR 'carcinoma, renal cell' OR 'clear cell renal cell carcinoma' OR 'kidney cell carcinoma' OR 'renal cell carcinoma') AND ('metastasis'/mj OR 'cancer metastasis' OR 'carcinoma metastasis' OR 'metastases' OR 'metastasic type' OR 'metastasis' OR 'metastatic cancer' OR 'metastatic cancers' OR 'metastatic carcinoma' OR 'metastatic disease' OR 'metastatic tumor' OR 'metastatic tumors' OR 'metastatic tumour' OR 'metastatic tumours' OR 'tumour metastasis'), 24.385
#2 'cancer immunotherapy'/mj OR 'tumor immunotherapy' OR 'tumour immunotherapy' OR 'cancer immunotherapy' OR 'immunotherapy, cancer' OR 'pembrolizumab'/mj OR 'keytruda' OR 'lambrolizumab' OR 'pembrolizumab' OR 'nivolumab'/mj OR 'nivolumab' OR 'opdivo' OR 'avelumab'/mj OR 'avelumab' OR 'bavencio' OR 'ipilimumab'/mj OR 'ipilimumab' OR 'strentarga' OR 'yervoy' OR 'atezolizumab'/mj OR 'atezolizumab' OR 'tecentriq' OR 'tecntriq' OR 'bevacizumab'/mj OR 'bevacizumab' OR 'bevacizumab beta' OR 'bevax', 156.746

#3 ('protein tyrosine kinase inhibitor'/mj OR 'protein tyrosine kinase inhibitor' OR 'tyrosine kinase inhibitor' OR 'tyrosine protein kinase inhibitor'), 48.614
#4 'sunitinib'/mj OR 'sunitinib' OR 'sunitinib malate', 23.715
#5 #2 OR #3, 200.362
#6 #4 AND #5, 12.996
#7 #1 AND #6, 2.529
#8 #1 AND #6 AND ([controlled clinical trial]/lim OR [randomized controlled trial]/lim), 225

Cochrane Central Register of Controlled Trials - CENTRAL (OVID)
#1 (clear cell renal cell carcinoma or renal cancer {Including Related Terms}).mp. [mp=title, original title, abstract, mesh headings, heading words, keyword}, 212
#2 protein tyrosine kinase inhibitor.af., 323
#3 tyrosine kinase inhibitor.af., 1797
#4 tyrosine protein kinase inhibitor.af., 1
#5 2 or 3 or 4, 1797
#6 cancer immunotherapy'/mj OR 'tumor immunotherapy' OR 'tumour immunotherapy' OR 'cancer immunotherapy' OR 'immunotherapy, cancer {Including Related Terms}, 1,186
#7 pembrolizumab' OR 'nivolumab' OR 'avelumab' OR 'ipilimumab' OR 'atezolizumab' OR 'bevacizumab' {Including Related Terms}, 4,609
#8 6 or 7,5,631
#9 5 or 8, 7.348
#10 sunitinib malate.af., 89
#11 sunitinib.af., 1,325
#12 10 or 11, 1325
#13 9 and 12, 337
#14 1 and 13, 22

ClinicalTrials
Carcinoma, Renal Cell OR Kidney Cancer |(pembrolizumab OR nivolumab OR avelumab OR ipilimumab OR Atezolizumab OR bevacizumab) AND sunitinib, 13

Section S2.

Figure S1. Risk of bias summary of RCTs with Cochrane Collaboration tool. Outcome: Overall survival in the favourable risk group.

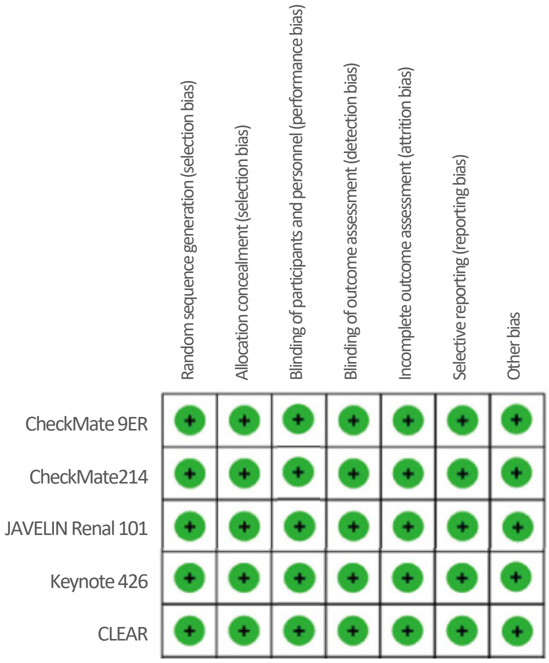

References

Choueiri TK, Powles T, Burotto M, Escudier B, Bourlon MT, Zurawski B, et al. Nivolumab plus Cabozantinib versus Sunitinib for Advanced Renal-Cell Carcinoma. New England Journal of Medicine. 4 de marzo de 2021;384(9):829-41.

Motzer RJ, Escudier B, McDermott DF, Frontera OA, Melichar B, Powles T, et al. Survival outcomes and independent response assessment with nivolumab plus ipilimumab versus sunitinib in patients with advanced renal cell carcinoma: 42-month follow-up of a randomized phase 3 clinical trial. Journal for immunotherapy of cancer. 2020;8(2).

Choueiri TK, Motzer RJ, Rini BI, Haanen J, Campbell MT, Venugopal B, et al. Updated efficacy results from the JAVELIN renal 101 trial: First-line avelumab plus axitinib versus sunitinib in patients with advanced renal cell carcinoma. Annals of Oncology. 2020 Aug;31(8):1030-1039

Powles T, Plimack ER, Soulières D, Waddell T, Stus V, Gafanov R, et al. Pembrolizumab plus axitinib versus sunitinib monotherapy as first-line treatment of advanced renal cell carcinoma (KEYNOTE-426): extended follow-up from a randomised, open-label, phase 3 trial. The Lancet Oncology. 2020;21(12):1563-73.

Motzer R, Alekseev B, Rha S-Y, Porta C, Eto M, Powles T, et al. Lenvatinib plus Pembrolizumab or Everolimus for Advanced Renal Cell Carcinoma. New England Journal of Medicine. 13 de febrero de 2021;384(14).

Figure S2. Risk of bias summary of RCTs with Cochrane Collaboration tool. Outcome: Progression free survival in the favourable risk group.

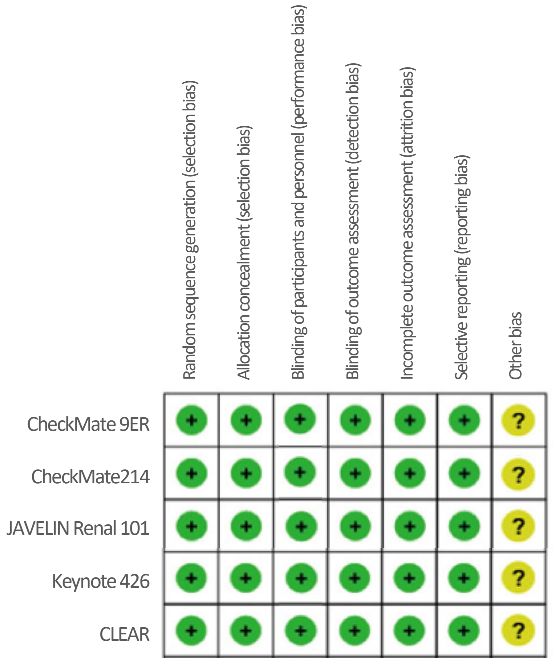

References

Choueiri TK, Powles T, Burotto M, Escudier B, Bourlon MT, Zurawski B, et al. Nivolumab plus Cabozantinib versus Sunitinib for Advanced Renal-Cell Carcinoma. New England Journal of Medicine. 4 de marzo de 2021;384(9):829-41.

Motzer RJ, Escudier B, McDermott DF, Frontera OA, Melichar B, Powles T, et al. Survival outcomes and independent response assessment with nivolumab plus ipilimumab versus sunitinib in patients with advanced renal cell carcinoma: 42-month follow-up of a randomized phase 3 clinical trial. Journal for immunotherapy of cancer. 2020;8(2).

Choueiri TK, Motzer RJ, Rini BI, Haanen J, Campbell MT, Venugopal B, et al. Updated efficacy results from the JAVELIN renal 101 trial: First-line avelumab plus axitinib versus sunitinib in patients with advanced renal cell carcinoma. Annals of Oncology. 2020 Aug;31(8):1030-1039

Powles T, Plimack ER, Soulières D, Waddell T, Stus V, Gafanov R, et al. Pembrolizumab plus axitinib versus sunitinib monotherapy as first-line treatment of advanced renal cell carcinoma (KEYNOTE-426): extended follow-up from a randomised, open-label, phase 3 trial. The Lancet Oncology. 2020;21(12):1563-73.

Motzer R, Alekseev B, Rha S-Y, Porta C, Eto M, Powles T, et al. Lenvatinib plus Pembrolizumab or Everolimus for Advanced Renal Cell Carcinoma. New England Journal of Medicine. 13 de febrero de 2021;384(14).

Table S1. GRADE table for survival from clinical trial data.

Date: February 2021

Question: What is the effect of combination therapy vs sunitinib on survival for advanced renal cell carcinoma in favorable risk patients?

Setting: Data from adults with advanced renal cell carcinoma in favourable risk patients included in clinical trials.

Quality assessment								No of subjects		Effect	Quality	Importance
**Outcome**	**No of studies**	**Design**	**Risk of bias**	**Inconsistency**	**Indirectness**	**Imprecision**	**Other considerations**	**I[a]**	**C[b]**	**Relative HR (95% CI)**		
Overall survival	5	randomised trials	not serious	not serious	not serious	serious [d]	None	2102	2004	1.07 [0.81, 1.41]	⊕⊕⊕◯ MODERATE	Critical
Progression-free survival	5	randomised trials	not serious	serious [c]	not serious	not serious	None	2102	2104	0.74 [0.46, 1.19]	⊕⊕⊕◯ MODERATE	Important

Explanations

[a] sunitinib

[b] immunotherapy: Pembrolizumab + axitinib, Avelumab + axitinib, Nivolumab + ipilimumab o cabozantinib, lenvatinib + pembrolizumab

[c] evidence of significant heterogeneity; I^2 >50%

[d] wide confidence interval
